# Epitranscriptome marks detection and localization of RNA modifying proteins in mammalian ovarian follicles

**DOI:** 10.1186/s13048-023-01172-8

**Published:** 2023-05-10

**Authors:** Karine Dubuc, Mathilde Marchais, Isabelle Gilbert, Alexandre Bastien, Karen E. Nenonene, Edward W. Khandjian, Robert S. Viger, Géraldine Delbes, Claude Robert

**Affiliations:** 1 Centre de Recherche en reproduction, développement et santé intergénérationnelle, Université Laval, Québec, QC Canada; 2 Département des sciences animales, Université Laval, Québec, QC Canada; 3 Département de psychiatrie et de neurosciences, Faculté de médecine, Université Laval, Québec, QC Canada; 4 Département d’obstétrique, gynécologie et reproduction, Faculté de médecine, Université Laval, Québec, QC Canada; 5 INRS- Armand-Frappier Santé Biotechnologie, Laval, QC Canada

**Keywords:** RNA methylation, Oocytes, Transzonal projections, Somatic cells, RNA modifying enzyme, Epitranscriptome

## Abstract

**Background:**

Most of the resources that support the early development of the embryo are stored in the oocyte. Clearing of maternal resources and activation of the embryonic genome to produce its own mRNA transcripts marks the maternal-to-embryo transition. Dependence on stored mRNA can last from a few hours to several days, depending on animal species. The mechanisms regulating stabilization and recruitment of stored maternal transcripts have not yet been described in full detail but are known to involve reversible polyadenylation and modulation of 3’UTR-mediated elements. RNA epigenetic modifications, new players in this field, have an important role in RNA regulation and stabilization.

**Results:**

The objectives of this study were first to determine if some of post-transcriptional methylation of stored mRNA is greater in oocytes than in somatic cells. We found that m^6^A, known to be the most prevalent and involved in various aspects of RNA metabolism and physiological functions, is particularly abundant in porcine oocyte mRNA compared to liver used as a somatic tissue reference. The second objective was to compare the epitranscriptome machinery, such as methyltransferases (“writers”), binding proteins (“readers”) and demethylases (“erasers”) catalyzing the different process, in follicles and oocytes of different mammalian species by immunofluorescence and confocal microscopy. The expression and localization patterns of these proteins differ between mice, pigs and cows ovaries and oocytes. m^5^C-associated proteins were generally less abundant. In contrast, m^6^A-associated proteins were expressed strongly during the early and late stages of folliculogenesis. Transzonal projections were found to contain more granules bearing the m^5^C mark in mice but both m^5^C and m^6^A methylation marks in association with mature oocytes of pigs and cows. Eraser proteins showed the greatest interspecies diversity in terms of distribution in the germinal tissues.

**Conclusions:**

So far, few studies have looked at the oocyte and ovarian epitranscriptomic profile. Our findings indicate that a hitherto unrecognized species-specific layer of transcript regulation occurs at the RNA level and might be consequential during the oocyte transcriptional silencing period.

**Supplementary Information:**

The online version contains supplementary material available at 10.1186/s13048-023-01172-8.

## Background

A central tenet of gene expression theory is that DNA-encoded information is first transcribed to RNA, an intermediary, transitory and short-lived molecule that is translated into protein as needed. However, oocytes display atypical RNA management, stocking molecules synthesized during oogenesis to support early development until embryonic genome activation [1]. Messenger RNA (mRNA) transcripts stored in oocytes exhibit an extended half-life that can be estimated in days rather than in minutes generally measured in somatic cells [2, 3].

Maternal RNA reserves ensure protein synthesis in the absence of transcriptional activity prior to genome activation [4, 5]. Transcriptional activity resumes differently depending on the species: 1-cell or 20 h post fertilization in mice [6], 4-cell or about 55 h post fertilization in pigs [7] and 8-cell or about 72 h post fertilization in cows [8-11].

The lifespan of maternal transcripts has not been measured directly but could in theory extend from final synthesis prior to DNA compaction in the oocyte to genome activation in the embryo. Compacted DNA is seen in oocytes still in the follicle. Based on the mean time for ovulation from this point plus fertilization and activation of embryo development, this lifespan can be estimated at 30 h in mice, 7 days in pigs and 10 days in cattle.

It is known that stabilization of maternal RNA involves removal of the poly(A) tail and packaging into ribonucleoprotein complexes [1]. In addition, specific sequences located in the 3’UTR have been shown to control stability [12]. The details of these mechanisms remain poorly understood and appear to differ greatly between species. Several RNA binding proteins and microRNA molecules have been shown to control the temporal destiny of transcripts but also the spatial distribution of mRNA in the oocyte cytoplasm [13, 14]. The influence of specific UTR sequences on maternal RNA stability is well documented, but spatial distribution has not been shown in mammalian oocytes [15].

Another type of post-transcriptional modification is RNA editing. Analogous to the epigenome, which is based primarily on 5-methylation of DNA cytidine groups (reviewed by Bird, 2002) [16], transcribed RNA is also modified by attachment of chemical groups [17]. Methylation is only one of more than 170 distinct chemical modifications that have been found on RNA molecules [17, 18]. The most abundant modification is N^6^-Methyladenosine (m^6^A), occurring mainly on mRNA and long non-coding RNA [19]. Enzymes that catalyze this reversible modification are called RNA modifying proteins or RMPs, of which three groups have been identified: writers, which catalyze the modification, erasers, which remove the marks, and readers, which recognize the modification and initiate a cellular response [20]. While the understanding of the function of the epitranscriptome has demonstrated its implication in various metabolic processes and diseases but its role in RNA stability and control of gene expression remains obscure.

We posit that some transcript labelling mechanism must exist and we hypothesized that different RNA methylation marks and process are involved in stabilizing stored maternal RNA. The aim of this study was therefore to compare the type and the abundance of RNA modifications in oocytes and somatic cells and to investigate if a relationship exists between the types of RMP present in oocytes of animals with different length of the transcriptional silence period. Modifications, namely m^6^A, were found abundantly in maternal RNA, as was the contingent of different RMPs, in some cases with species specific distributions.

## Methods

### Animals

All animals used in this study were handled according to the guidelines of the Canadian council on animal care and manipulation of animals used in research. Mouse experiments were carried out on 8-weeks old animals in accordance with protocols approved by the Comité de Protection des Animaux du Centre Hospitalier Universitaire de Québec. Bovine ovaries from 2–5 years old dairy cows and porcine ovaries and liver tissues from 5 months old gilts were collected from a local slaughterhouse.

All chemicals and enzymes, unless otherwise specified, were purchased from Sigma-Aldrich (Oakville, ON, Canada).

### RNA isolation

Total RNA from oocytes, cumulus and granulosa cells were extracted with the Picopure RNA Isolation Kit (Thermo Fisher Scientific, Mississauga, ON, Canada) including on-column DNAse I digestion (Qiagen, Mississauga, ON, Canada) in accordance with the manufacturer’s instructions. Cumulus and granulosa cells were homogenized beforehand using QIAshredder spin-columns (Qiagen) and ovarian and liver tissues were homogenized using a Ruptor bead mill as recommended by the manufacturer (VWR International, Mississauga, ON, Canada). Total RNA was extracted from homogenates using TRIzol (Thermo Fisher Scientific) then isolated using a Picopure kit. Total RNA integrity and concentration were evaluated on a 2100-Bioanalyzer (Agilent Technologies, Palo Alto, CA, USA) using the RNA 6000 Nano Kit (Agilent Technologies). Samples with a RIN greater than 7 were chosen.

### RNA enzymatic digestion and LC–MS/MS analysis

RNA was converted to mono-ribonucleotides as described previously [21, 22]. Briefly, 1 µg of RNA was hydrolyzed for 3 h with 0.2 U of nuclease P1 in 60 µl of ammonium acetate buffer (pH 5.3) at 50 °C followed by 0.04 U of phosphodiesterase I (Thermo Fisher Scientific) for 2 h then 2 U of alkaline phosphatase for 2 h (both at 37 °C). Enzymes and buffers were removed using Nanosep 3 K columns (VWR International) centrifuged at 5000 × g for 30 min. Concentrated nucleotides were eluted in two 20 µl fractions of nuclease-free water for a total of 40 µl, dried using a speed-vac and kept at -80 °C until LC–MS/MS analysis as described previously [22]. Experiments were performed in triplicate. Experimental effects on nucleoside methylation were assessed by one-way ANOVA and the Tukey multiple comparisons test. Treatment means were considered significantly different when *p* < 0.05.

### Ovary section immunohistofluorescence

Ovarian tissue (15 mm diameter, 5 mm thick) was fixed in 4% (v/v) paraformaldehyde in phosphate-buffered saline (PBS, Thermo Fisher Scientific) overnight at room temperature then dehydrated in ethanol at increasing concentrations and embedded in paraffin. Sections 7 µm thick were cut on a microtome, mounted in series on positively charged glass slides, dewaxed with xylene and rehydrated by washing with ethanol at concentrations decreasing to zero in distilled water. The slides were then processed in heat-induced epitope retrieval buffer (HIER, Agilent) in a pressure cooker at 95 °C for 20 min as per the manufacturer’s instructions (Biocare medical, Concord, CA, USA). Slides were cooled for about 5 min at room temperature then washed 3 times in PBS for 5 min each. Non‐specific binding was prevented by blocking with 5% donkey serum diluted in Tris-buffered saline with Tween 20 (TBST) for 1 h before applying the primary anti-rabbit or anti-chicken antibody diluted in TBST or the corresponding secondary antibody as a negative control (supplementary Fig. S1; antibodies are listed in supplementary Table S1). The slides were kept overnight at 4 °C in a humidified chamber then washed 3 times in TBST for 10 min each with gentle agitation and exposed for 1 h to the secondary antibody (diluted 1:1000 in TBST containing 20% bovine serum albumin) at room temperature with gentle agitation and shielding from light. DNA was stained using Hoechst 33,342 (Thermo Fisher Scientific). After three washes in PBS/TBST, slides were mounted in SlowFade Diamond Antifade Mountant (Thermo Fisher Scientific).

### Whole-mount oocyte immunofluorescence

Except for primary antibody applications, all steps below were performed at room temperature. Oocytes were fixed in 4% (v/v) paraformaldehyde in PBS-polyvinyl alcohol (PBS-PVA) for 15 min followed by three 5-min washes in PBS-PVA then permeabilized in Triton X-100 in TBST (1:200) for 15 min, blocked for 1 h with donkey serum (Sigma-Aldrich) diluted in TBST (1:20) and contacted with the primary antibody (in TBST with 20% BSA) overnight at 4 °C. Application of secondary antibody alone served as a negative control (supplementary Fig. S2). The oocytes were then washed 3 times in TBST (10 min each) and exposed for 1 h to secondary antibody (donkey anti-rabbit 488 or donkey anti-chicken 488, Thermo Fisher Scientific, diluted 1:1000 in TBST). DNA and actin (cytoskeleton) were stained respectively with Hoechst 33,342 (1:1000 in TBST) and Acti-Stain 555 (7:1000 in TBST) for 1 h. Oocytes were then washed 3 times in PBS-PVA (10 min each) and mounted in PBS-PVA.

### Ovary and oocyte imaging

Z-stack images of oocytes and ovaries were captured using a Zeiss LSM700 Confocal microscope (Carl Zeiss Canada, Toronto, ON, Canada) fitted with a 40X water-immersion Zeiss objective and Zen Black acquisition software. The images were analyzed using Fiji ImageJ [23]. Secondary-only controls are shown in supplementary data. At least four ovary section slides and pools of 15 whole-mount oocytes from three different animals were examined.

## Results

### Identification and measurement of RNA modifications by LC–MS/MS analysis

**Fig. 1:**
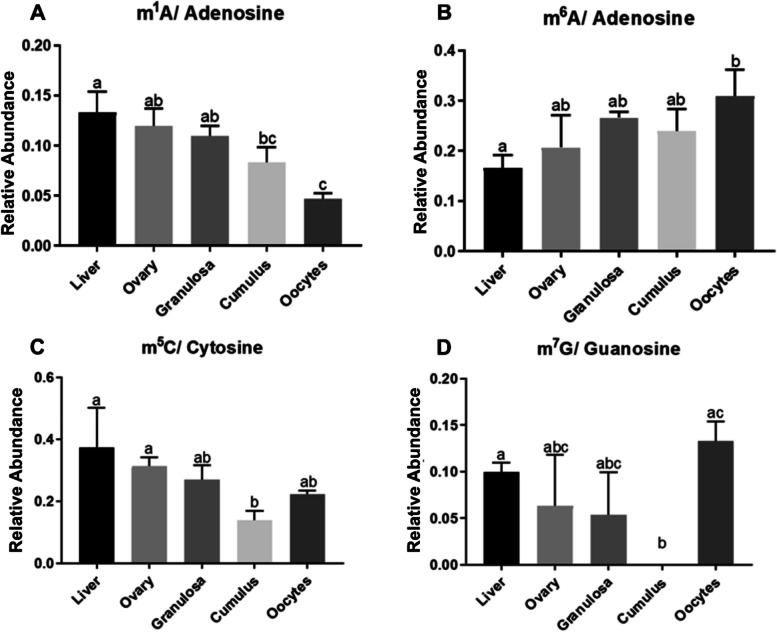
Relative expression of modifications of porcine somatic and germinal cell RNA, based on mass spectrometry (**A**) N^1^-methyladenosine, **B** N.^6^-methyladenosine, **C** 5-methylcytidine, **D** 7-methylguanosine. Means denoted by a different letter indicate significant differences between tissues (*p* < 0.05)

Methylated ribonucleosides, namely N^1^-methyladenosine, N^6^-methyladenosine, 5-methylcytosine and 7-methylguanosine (respectively m^1^A, m^6^A, m^5^C and m^7^G) in total RNA of oocytes, cumulus cells, granulosa cells, ovarian and liver tissue from pigs were detected quantitatively using LC–MS/MS. Liver was used as a somatic cell reference since mRNA is rarely stable in this tissue. As shown in Fig. 1 (A, B), RNA m^1^A was less abundant in oocytes than in liver, ovary and granulosa cells whereas m^6^A was more abundant in oocyte compared to liver. In the case of m^5^C and m^7^G, cumulus cells stand out with a low abundance of these molecules (Fig. 1C, D). In all tissues tested, the most abundant post-transcriptional modifications of RNA were m^6^A and m^5^C.

### Detection of RNA modifying proteins

Using immunofluorescence and confocal microscopy, RMP writers, erasers and readers in follicles and oocytes from mice, porcine and bovine tissues were examined.

### Detection of writer proteins during folliculogenesis

**Fig. 2:**
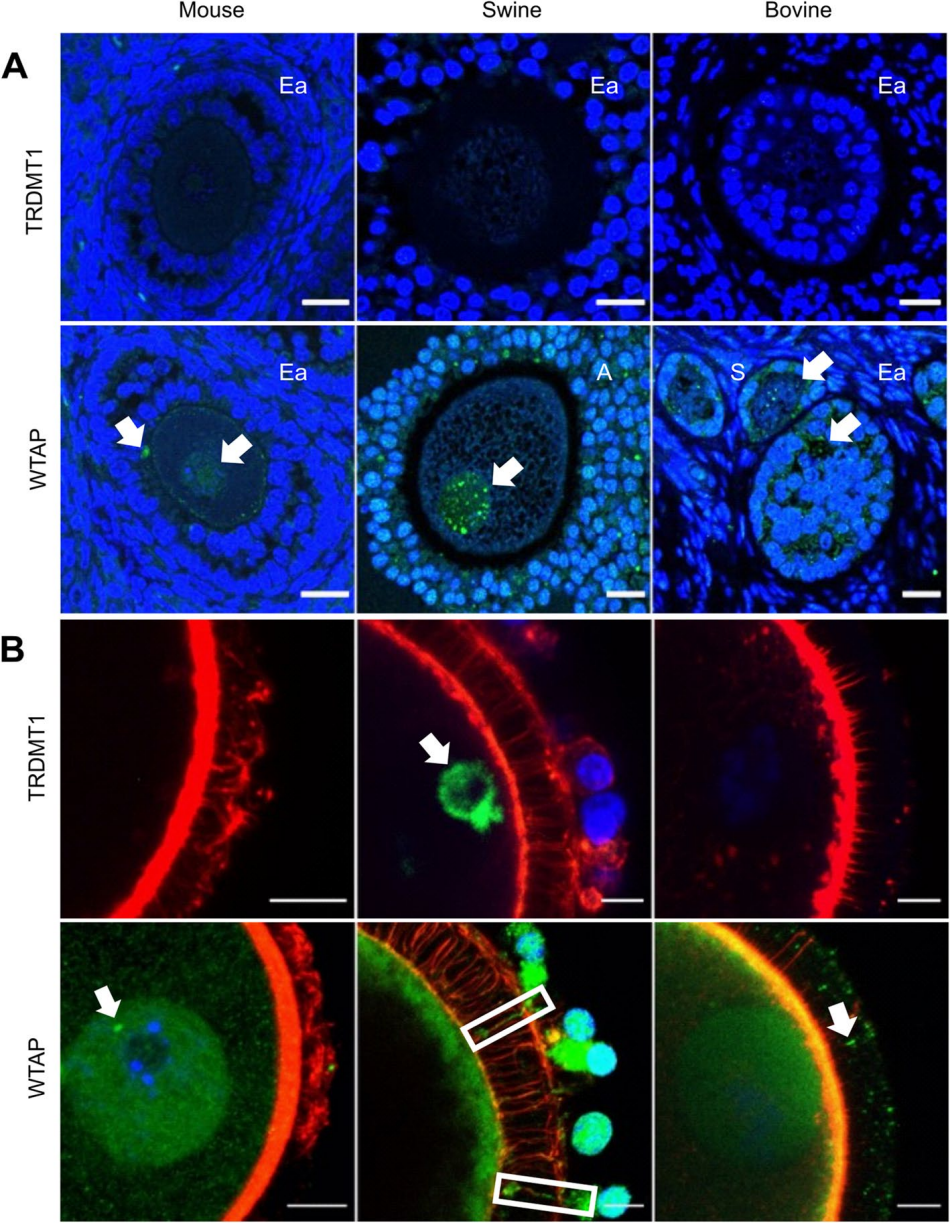
Representative confocal Z-stack images showing TRDMT1 and WTAP writer protein distribution (green) in murine, porcine and bovine germinal tissue obtained using fluorescent antibodies (green). **A** Sections of ovary showing secondary (S), early antral (Ea) and antral (A) follicle stages (bar = 20 μm). **B** Whole oocyte mounts (bar = 10 μm). Actin filaments of TZPs were stained with Acti-stain 555 phalloidin (red) and DNA with Hoechst 33,342 (blue)

TRDMT1 (aka DNMT2) or tRNA aspartic acid methyltransferase 1 catalyzes methylation of cytosine. It is also slightly active as a DNA methyl transferase. This writer protein was barely detectable in early antral follicles of mice, pigs and cows and in mouse and cow oocytes (Fig. 2A and B). It was detected in the nucleus of porcine mature oocytes (Fig. 2B).

In contrast, Wilms Tumor-Associated Protein 1 (WTAP), which produces m^6^A, was detected in mouse oocytes in pre-antral follicles, especially in the nucleus and around the zona pellucida. It appeared in the porcine oocyte nucleus (spots in Fig. 2A) and in follicular cells but not the nucleus of bovine oocytes (Fig. 2A, lower panel). During the later stages of follicular development, WTAP was found in the cytoplasm and nucleus of mature murine and bovine oocytes. In both large animals but not in mice, it was also localized throughout the network of transzonal projections (Fig. 2B, lower panel).

### Detection of reader proteins

**Fig. 3:**
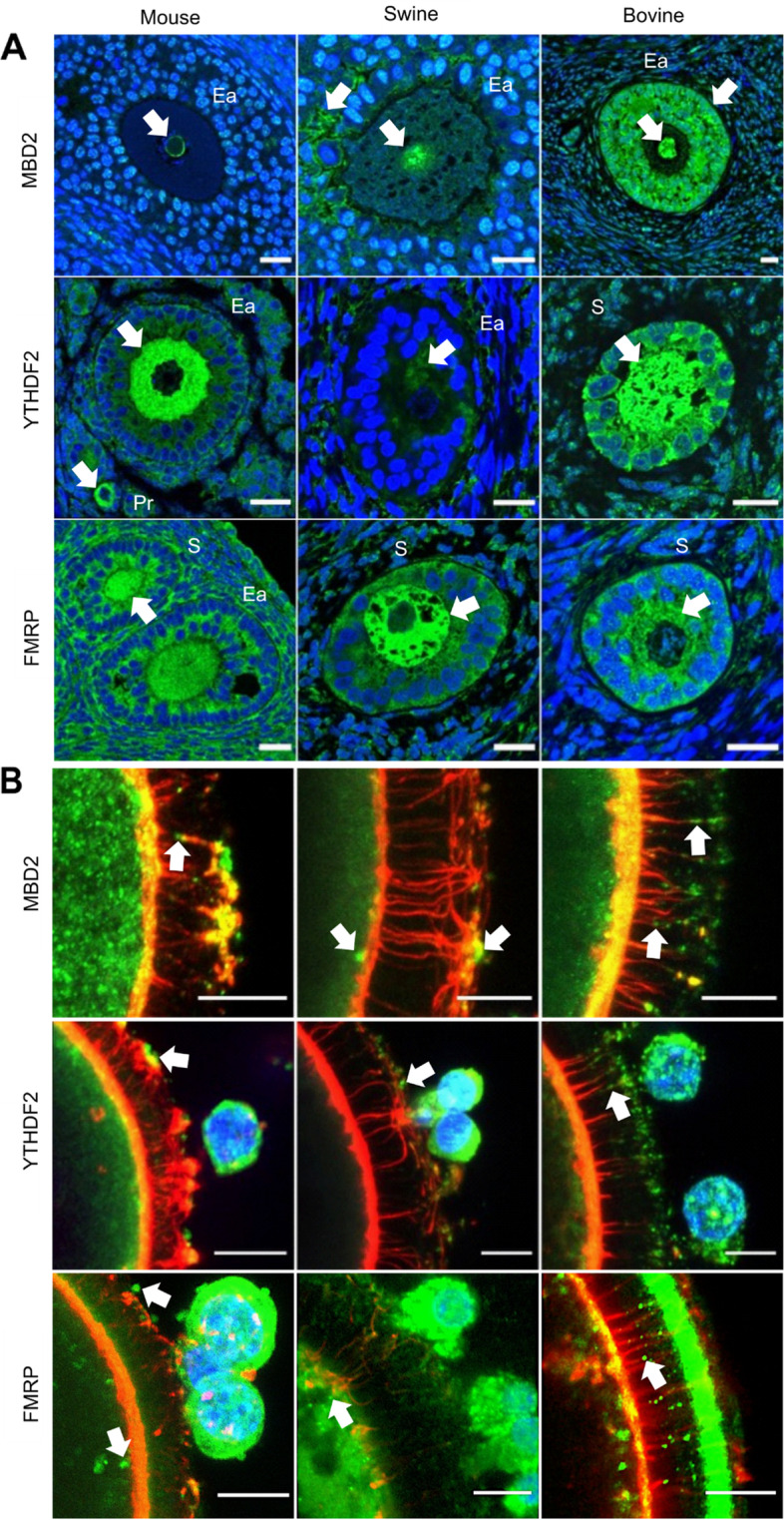
Representative confocal Z-stack images of MBD2, YTHDF2 and FMRP reader protein distribution (green) in murine, porcine and bovine germinal tissue. **A** Sections of ovary showing primary (Pr), secondary (S) and early antral (Ea) follicle stages (bar = 20 μm). **B** Whole oocyte mounts (bar = 10 μm). Actin filaments of TZPs were stained with Acti-stain 555 phalloidin (red) and DNA with Hoechst 33,342 (blue)

A family of proteins binds to methyl-CpG domains on DNA [24]. One of these, called Methyl-CpG Binding Domain Protein 2 (MBD2), has a high affinity for m^5^C-modified RNA [25, 26]. This protein was detected in the nucleus of early antral follicles in all species, but curiously only at the periphery of the oocyte nucleus in mice (Fig. 3A). It was more abundant both in the nucleus and the first layers of porcine cumulus cells and even more in the oocyte nucleus and cytoplasm, and in bovine cumulus cells. In fully mature oocytes, it was detected in the zona pellucida and at the end of transzonal projections in all three species, perhaps less in pigs (Fig. 3B).

Another reader protein, YTH N6-Methyladenosine RNA Binding Protein F2 (YTHDF2) member of the YTH domain family, recognize and bind to m^6^A (N6-methyladenosine) to regulate mRNA stability. YTHDF2 was distributed throughout the oocyte cytoplasm in all three species (Fig. 3A, middle panels) and was detectable in granulosa/cumulus cells and in the cytoplasm of mature murine or bovine but not porcine oocytes. Cumulus cells of all three species and the edge of murine and bovine zona pellucidae also contained YTHDF2, whereas transzonal protections contained very little (Fig. 3B, middle panels).

The RNA-binding protein FMRP (known as Fragile X Mental Retardation Protein or Fragile X Multifunctional RNA-binding Protein) is involved in shuttling transcripts and regulating their docking on ribosomes [27, 28]. Its binding is enhanced by m^6^A [29]. It was expressed strongly in early follicles of all three species, in mouse ovarian somatic cells and inside porcine and bovine oocyte follicular compartments as well as the oocyte cytoplasm (Fig. 3A). It was detected in antral follicles of all three species, in foci located under the zona pellucida and inside cumulus cells (Fig. 3B, lower panel). FMRP-containing granules were absent in murine and porcine transzonal projections but abundant near the cumulus side of the projections.

### Detection of RNA methylation eraser proteins

**Fig. 4:**
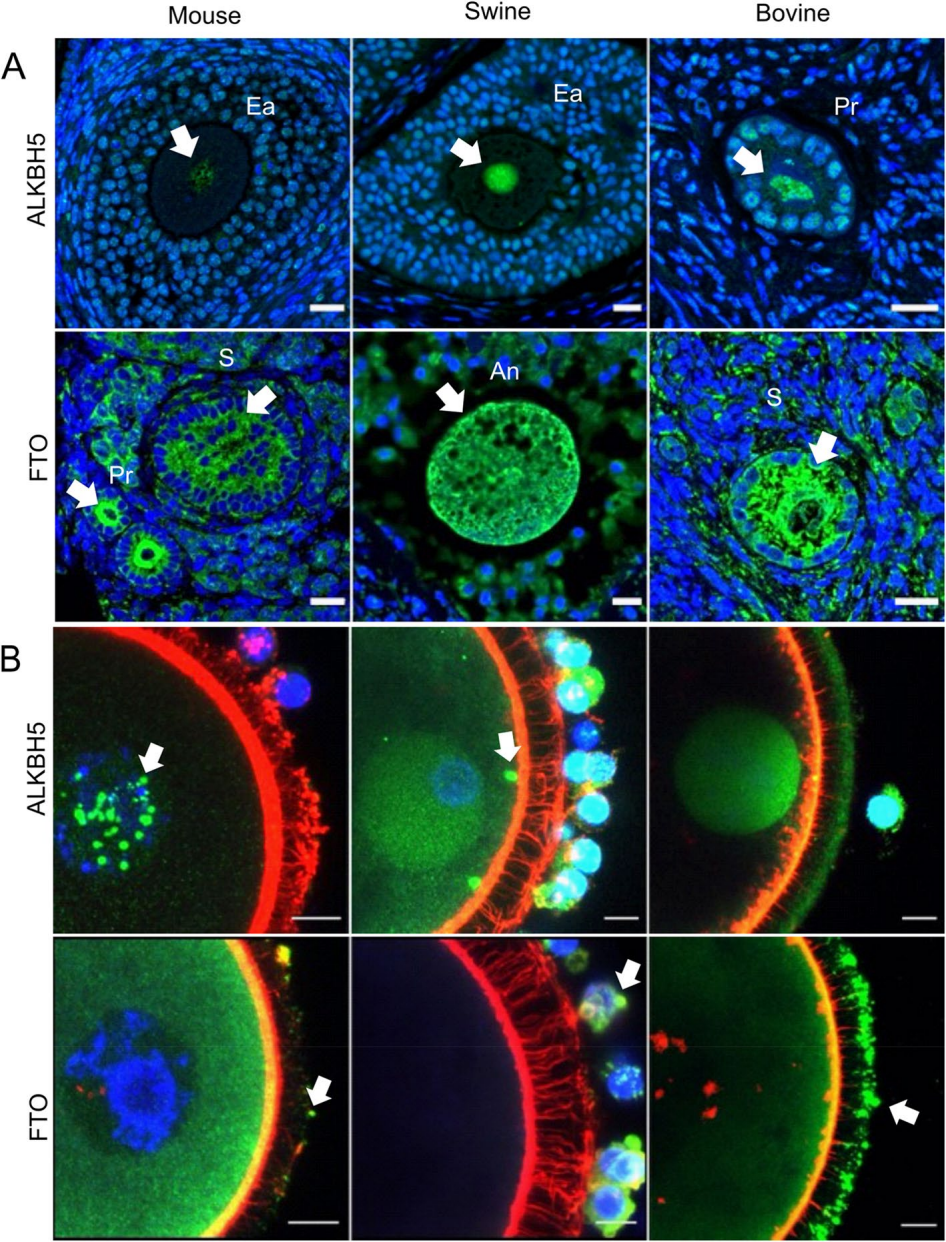
Representative confocal Z-stack images of ALKBH5 and FTO eraser protein distribution (in green) in murine, porcine and bovine germinal tissue. **A** Sections of ovary showing primary (Pr), secondary (S) and early antral (Ea) and antral (An) follicle stages (bar: 20 μm). **B** Whole oocyte mounts (bar: 10 μm). Actin filaments of TZPs were stained with Acti-stain 555 phalloidin (red) and DNA with Hoechst 33,342 (blue)

Chemical marks imprinted on RNA by writer proteins can be erased by the action of demethylases such as AlkB homolog 5, RNA Demethylase (ALKBH5) or by FTO Alpha-Ketoglutarate Dependent Dioxygenase (FTO). In early follicles of all three species, ALKBH5 was found mostly in the oocyte nucleus (Fig. 4A). In antral follicles of mice, it appeared only in the gamete nucleus in a dotted pattern having no relationship to DNA (Fig. 4B). In the case of pigs and cows, it appeared in a diffuse state in the nuclear compartment but also in large foci in the subcortical region under the zona pellucida and was expressed strongly in cumulus cells. The distribution of FTO was broader, with a strong expression in the cytoplasm of early oocytes in all three species, finely dispersed in pig oocytes at a later follicular stage, and granular near the outer end of transzonal projections (the cumulus cell side of the zona pellucida) in pigs and cows. FTO granules inside transzonal projections were seen only in bovine oocytes. Table 1 provides a summary of the localization of the molecules detected.

**Table 1:** Localisation of RNA modifying proteins protein in early antral follicles and mature oocytes

Enzyme type	Germinal tissue type					
	**Early antral**			**Mature oocyte**		
	**Mouse**	**Pig**	**Cow**	**Mouse**	**Pig**	**Cow**
**Writer**						
TRDMT1	n.d	n.d	n.d	n.d	oon	n.d
WTAP	oon, zp	oon	fc	oon, ocy, cc	ocy, zp, tpi, tpe, cc	oon, ocy, zp, tpi, tpe, cc
**Reader**						
MBD2	oon	oon, ocy, cc	oon, gc	oon, ocy^a^, zp, tpe, cc	oon, ocy, zp, tpe, cc	oon, ocy, zp, tpi, tpe, cc
YTHDF2	ocy, fc	ocy^b^	ocy, fc	ocy, zp, tpe, cc	tpze, cc	ocy, tpi, tpe^1^, cc
FMRP	ocy, cc, fc^2^	ocy, cc, fc	ocy, cc, fc	ocy, zp, tpe, cc	ocy, zp, tpi, tpe, cc	zp, tpi, tpe, cc
**Eraser**						
ALKBH5	oon	oon	oon	oon^c^	oon, ocy, zp^d^, cc	oon, tpe, cc
FTO	ocy, fc^e^	ocy, fc	ocy, fc	ocy, zp, tpe, cc	cc	ocy, zp, tpi, cc

*n.d.* not determined, *oon* oocyte nucleus, *ocy* oocyte cytoplasm, *fc* follicular cells, *zp* zona pellucida, *tpi* transzonal projection insides, *tpe* transzonal projection ends, *cc* cumulus cells, *gc* granulosa cells; ^a^high intensity; ^b^low intensity; ^c^spotty; ^d^large foci; ^e^primordial

## Discussion

Oocytes accumulate RNA reserves to sustain protein synthesis during the transcriptional silence that reigns from the moment the genome condenses in the immature oocyte until activation of the embryonic genome [5, 30]. The factors determining which stored mRNA transcripts are active during mammalian early development remain largely unknown but studies of *Drosophila* and *Xenopus* have shown clearly that mRNA is not stored randomly in the oocyte cytoplasm [31, 32]. Furthermore, it has been shown that maternal reserves need to be broken down for embryonic genome activation to occur [33, 34]. Their extended lifespan and active decay mechanisms in mice nevertheless indicate clearly that mRNA stability is crucial for constituting the reserves and seeing to their timely clearing.

The finding that m^6^A modification of RNA is more frequent in oocytes than in liver cells at least in pigs has interesting implications for maternal transcripts, since it has been demonstrated that an abundance of m^6^A near the stop codon in 3’UTRs affects polyadenylation site selection [35]. Another study in pigs has detected a greater abundance of m^6^A marks in the 5’ and 3’ UTR gene coding regions [36]. Moreover, it has found that the m^6^A is abundant in granulosa cells and this mark is greater in small (< 3 mm) compared to large follicles (> 5 mm) demonstrating a potential role of m^6^A during folliculogenesis through the modulation of specific transcript [36]. Since reproductive organs like ovaries exhibits a rate of aging that is much faster than other somatic, it has also been shown that m^6^A associated gene in granulosa cells from aging women, an increased number of m^6^A methylated genes compared to younger female group [37]. Differences in polyadenylation patterns have been associated with differences in transcript stability, export, translation and localization [38]. Transcriptional silencing of the X chromosome has been associated with the presence of at least 78 m^6^A marks throughout the long non-coding RNA of XIST [39]. Taken together these results provide novel insights of m^6^A in female fertility and its implication in various functions from transcript stability to folliculogenesis and ovarian functions through aging.

Just like on DNA, epigenomic marks on RNA are due to enzymatic activities that transfer, recognize or remove them. Some of these enzymes have been associated with infertility of one type or another. TRDMT1 (DNMT2) combined with NOL1/NOP2/SUN as partner enzymes has been shown to catalyze writing of m^5^C modifications [40]. Although we detected m^5^C by mass spectrometry, expression of TRDMT1 was very low in all species except for some in the nucleus of porcine oocytes. The presence of TRDMT1 in the nucleus was expected since it has CpG DNA methylation activity, which plays a key role in oogenesis and embryonic development [41, 42]. Cytosine methylation by TRDMT1 is important for the stabilization of tRNA [43]. In *Drosophila*, 5-hydroxymethylcytosine is found on polyadenylated RNA and promotes mRNA translation in different physiological processes including embryogenesis [44]. In the present study, WTAP was found in all three mammalian species. This m^6^A writer is known to play a role in embryogenesis and tissue differentiation in the zebra fish [45] through transcriptional and post-transcriptional regulation of various genes. It is localized in the nucleoplasm and in pre-mRNA-rich speckles, and co-localizes partially with splicing factors [45, 46]. In mice, *wtap* knock-out becomes lethal during early embryogenesis [47].

The reader proteins MBD2, YTHDF2 and FMRP were detected in the cytoplasm of early and mature oocytes in all three species. MBD2 proteins offer different localization in mice and bovine early embryo stages and MD2 knockout mice is not known to lead to gross abnormalities and animals remain fertile [24, 48]. However, YTHDF2 appears to be required for oocyte quality and zygotic development in mammals and regulates transcript dosage during oocyte maturation in mice [49]. YTHDF2 with YTHDC2 have been shown to be crucial for meiotic initiation and progression in mice female germ cells [50, 51]. *FMR1* is located on the X chromosome and codes for an RNA-binding protein [52]. FMRP (the protein produced by *FMR1*) is considered as an m^6^A reader since it controls the export, the stability and the translation of methylated RNAs [29, 53-55]. Binding to m^6^A appears to be sequence-dependent, and possibly involve interaction with YTHDF [55]. *FMR1* dysregulation is known to increase the risk of primary ovarian insufficiency leading to premature menopause [52]. How FMRP might cause primary ovarian insufficiency is still unclear, but its association is consistent with the role of RNA binding proteins that recognize methylation marks as essential elements of ovarian function.

The greatest differences between species were observed for eraser enzymes, both in terms of abundance and localisation. FTO was the most abundant in the oocyte cytoplasm and most abundant in mice. ALKBH5 and FTO are both essential for fertility, the former being involved in mRNA export and RNA metabolism as well as important for spermatogenesis [56] and the latter playing some role in preventing premature ovarian insufficiency disease [57]. It has also been shown that both are localized in the nucleus of eukaryotic cells [19, 58], although we found that FTO was most abundant in the oocyte cytoplasm. FTO can also be an eraser for N^6^,2’-O-dimethyladenosine (m^6^A_m_), a post-transcriptional modification that occurs on the first nucleotide on mRNA following the m^7^G cap [59]. The reversible m^6^A_m_ mark stabilizes mRNA [60]. Our observations of nucleoside modifications and the abundance of the associated RMPs are consistent with this important role of m^6^A during oogenesis. The differences between species in terms of FTO are consistent with the timing required for the breakdown of maternal reserves.

We have reported previously that in cattle, transzonal projections connecting cumulus cells to the oocyte act like synapses and can harbor ribonucleoprotein granules or transfer these and other complex materials to the oocyte [61, 62]. RNA thus transferred would add to the maternal reserves. In this study, we showed that m^6^A writer WTAP is detectable in transzonal projections of both large mammals but not in those of mice. The three readers were found inside projections in all three species, whereas FTO was only in the case of cattle. This is consistent with observed cross-species differences in the duration of transzonal attachment to the *oolemma*, for example, persisting during maturation in mice, rats, rabbits and humans but disconnecting and retracting immediately after germinal vesicle breakdown in cattle [63].

The mechanisms underlying RNA shuttling through transzonal projections are still largely unknown, but we and others have described the role played by FMRP in RNA granules transport in mouse neurons [28]. N^6^-methyladenosine pulldown has allowed identification of FMRP, FXR1P and FXR2P as new readers [64]. Moreover, in mouse embryonic stem cells, FMRP also shares targets with YTHDF1 and promotes nuclear export of transcripts containing m^6^A residues [29, 64]. These reports all corroborate the involvement of RNA modifying enzymes in transcript stability, transport and translational control. 

## Conclusions

Three animal species were compared in an attempt to reveal the role of methylation in the persistence of maternal mRNA during mammalian early development. The longest transcriptional silence period and the earliest disconnection of transzonal projections following meiosis resumption are observed in bovine oocytes. Murine oocytes have the shortest transcriptional silence and are the least dependent on transcript stability and storage. Known modifiers of RNA nucleoside bases were very abundant in oocytes of all three species from the early to antral follicle stages. Species-associated differences in the distribution of these enzymes were observed, notably for the m^6^A eraser FTO. Increased presence of RNA modifying enzymes in transzonal projections was associated with greater dependence on RNA stability. This study provides support for an important role played by the epitranscriptome in the management of maternal RNA stored in mammalian oocytes.

## Supplementary Information

**Additional file 1:Table S1.** Description of the antibodies used for immunofluorescence in this study.

**Additional file 2:Figure S1.** Ovarian section in paraffin, negative control without primary antibody, showing no specific staining by the secondary antibody. DNA is stained with Hoescht 33342 (blue). Bar = 20 µm.

**Additional file 3:Figure S2.** Oocyte whole mount, negative control without primary antibody, showing no specific staining by the secondary antibody. DNA is stained with Hoescht 33342 (blue) and actin with Acti-stain 555 phalloidin (red). Bar = 20 µm (same magnification was used for swine and bovine).

**Additional file 4:**Stage and diameter of the follicles used in this study and the corresponding chromatin stage and transcription activity reported in the literature[65-68].

## Data Availability

The datasets used during the current study are available from the corresponding author on reasonable request.

## Abbreviations

ALKBH5: AlkB homolog 5, RNA Demethylase
FMR1: Fragile X Messenger Ribonucleoprotein 1
FMRP: Fragile X Mental Retardation Protein or Fragile X Multifunctional RNA-binding Protein)
FTO: FTO Alpha-Ketoglutarate Dependent Dioxygenase
LC–MS-MS: Liquid Chromatography with tandem mass spectrometry
mRNA: Messenger RNA
m6A: N6-Methyladenosine
m1A: N1-methyladenosine
m5C: 5-Methylcytosine
m7G: 7-Methylguanosine
MBD2: Methyl-CpG Binding Domain Protein 2
RMP: RNA modifying protein
TRDMT1: TRNA aspartic acid methyltransferase 1
UTR: Untranslated region
WTAP: Wilms Tumor-Associated Protein 1
YTHDF2: YTH N6-Methyladenosine RNA Binding Protein F2
