# Supplementary material for: Epitranscriptome marks detection and localization of RNA modifying proteins in mammalian ovarian follicles
Source: J Ovarian Res. 2023 May 10;16:90. doi: 10.1186/s13048-023-01172-8 (PMC10170753; doi:10.1186/s13048-023-01172-8)
Supplement: Supplementary file 1 — Additional file 1:Table S1. Description of the antibodies used for immunofluorescence in this study. [file 13048_2023_1172_MOESM1_ESM.docx]

# Supplementary Data

**Table S1.** Description of the antibodies used for immunofluorescence in this study

| Antibody | Dilution | Source |
| --- | --- | --- |
| Rabbit anti-DNMT2 | 1/100 | Abcam |
| Rabbit anti-WTAP | 1/50 | Abcam |
| Rabbit anti-MBD2 | 1/250 | Novus Biologicals |
| Rabbit anti-YTHDF2 | 1/50 | Proteintech |
| Chicken anti-FMRP #C10 | 1/100 | (El Fatimy et al., 2012 [69]) |
| Rabbit anti-ALKBH5 | 1/100 | Sigma-Aldrich |
| Rabbit anti-FTO | 1/200 | OriGene |
| Alexa-fluor 488 anti-rabbit | 1/1000 | Invitrogen |
| Alexa-fluor 488 anti-chicken | 1/1000 | Biotium |
