# Supplementary material for: Epitranscriptome marks detection and localization of RNA modifying proteins in mammalian ovarian follicles
Source: J Ovarian Res. 2023 May 10;16:90. doi: 10.1186/s13048-023-01172-8 (PMC10170753; doi:10.1186/s13048-023-01172-8)

**Fig. S1**. Ovarian section in paraffin, negative control without primary antibody, showing no specific staining by the secondary antibody. DNA is stained with Hoescht 33342 (blue). Bar = 20 µm


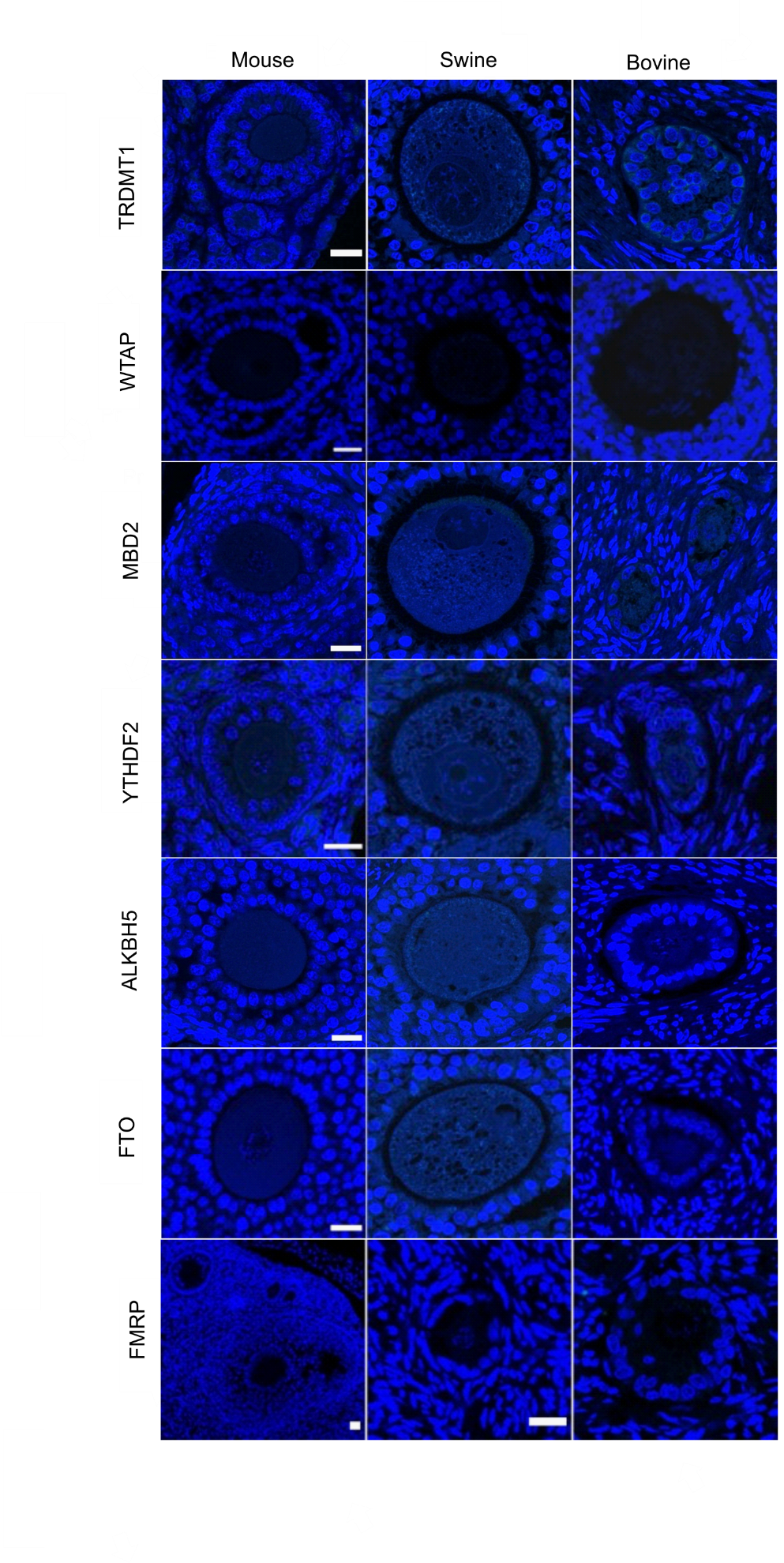

Supplement: Supplementary file 2 — Additional file 2:Figure S1. Ovarian section in paraffin, negative control without primary antibody, showing no specific staining by the secondary antibody. DNA is stained with Hoescht 33342 (blue). Bar = 20 µm. [file 13048_2023_1172_MOESM2_ESM.docx]
