# Supplementary material for: Epitranscriptome marks detection and localization of RNA modifying proteins in mammalian ovarian follicles
Source: J Ovarian Res. 2023 May 10;16:90. doi: 10.1186/s13048-023-01172-8 (PMC10170753; doi:10.1186/s13048-023-01172-8)
Supplement: Supplementary file 3 — Additional file 3:Figure S2. Oocyte whole mount, negative control without primary antibody, showing no specific staining by the secondary antibody. DNA is stained with Hoescht 33342 (blue) and actin with Acti-stain 555 phalloidin (red). Bar = 20 µm (same magnification was used for swine and bovine). [file 13048_2023_1172_MOESM3_ESM.docx]

**Fig. S2**. Oocyte whole mount, negative control without primary antibody, showing no specific staining by the secondary antibody. DNA is stained with Hoescht 33342 (blue) and actin with Acti-stain 555 phalloidin (red). Bar = 20 µm (same magnification was used for swine and bovine)
