# Supplementary material for: Epitranscriptome marks detection and localization of RNA modifying proteins in mammalian ovarian follicles
Source: J Ovarian Res. 2023 May 10;16:90. doi: 10.1186/s13048-023-01172-8 (PMC10170753; doi:10.1186/s13048-023-01172-8)
Supplement: Supplementary file 4 — Additional file 4:Stage and diameter of the follicles used in this study and the corresponding chromatin stage and transcription activity reported in the literature[65-68]. [file 13048_2023_1172_MOESM4_ESM.docx]

**Table 2.** Stage and diameter of the follicles used in this study and the corresponding chromatin stage and transcription activity reported in the literature

| **Factor** | **Follicular tissue type** | | | | | | |  | |
| --- | --- | --- | --- | --- | --- | --- | --- | --- | --- |
|  | **Early antral** | | | **Mature oocyte** | | | | **Ref** | |
|  | **Mouse** | **Pig** | **Cow** | **Mouse** | **Pig** | **Cow** |  | |  |
| **Follicle diameter (mm)** | 0.1–0.2 | < 0.001 | 0.5–2 | 0.300–350^1^ | 1–3^2^ | 2–6^2^ | Tan *et al.*, 2009 [30];  Xiao *et al.*, 2015 [66] | |  |
| **GV stage** |  | 0/1 | 0/1 | 1/2/3 | 1/2/3 | 1/2/3 | Tan *et al.*, 2009 [30]; Bui *et al.*, 2008 [67] | |  |
| **Chromatin configuration** | SN (84%) | NSN/SN | NSN | SN (100%) | SN/SN/SN | N, C, SN (22–45%) | Tan *et al.*, 2009 [30] | |  |
| **Transcription activity** | no | yes/no | yes | no | no/no/low | low/low/no | Luciano *et al.*, 2014 [68] | |  |

^1^ antral; ^2^ medium antral; SN : surrounded nucleolus; NSN : non-surrounded nucleolus.
